# Refining of cancer-specific genes in microsatellite-unstable colon and endometrial cancers using modified partial least square discriminant analysis

**DOI:** 10.1097/MD.0000000000041134

**Published:** 2024-12-27

**Authors:** Woong Na, Sung Hak Lee, Seunghee Lee, Jong-Seok Kim, Seung Yun Han, Yong Min Kim, Mihye Kwon, Young Soo Song

**Affiliations:** a Department of Pathology, H Plus Yangji Hospital, Seoul, South Korea; b Department of Hospital Pathology, College of Medicine, The Catholic University of Korea, Seoul, South Korea; c KYMERA (Konyang Medical Data Research Group), Konyang University Hospital, Daejeon, South Korea; d Myunggok Medical Research Center (Institute), College of Medicine, Konyang University, Daejeon, South Korea; e Department of Anatomy, College of Medicine, Konyang University, Daejeon, South Korea; f Department of Pathology, College of Medicine, Konyang University, Daejeon, South Korea; g Department of Internal Medicine, College of Medicine, Konyang University, Daejeon, South Korea.

**Keywords:** colon cancer, differentially expressed gene, endometrial cancer, gene expression profiling, microsatellite instability, partial least square discriminant analysis

## Abstract

Despite similarities in microsatellite instability (MSI) between colon and endometrial cancer, there are many clinically important organ-specific features. The molecular differences between these 2 MSI cancers are underexplored because the usual differentially expressed gene analysis yields too many noncancer-specific normally expressed genes. We aimed to identify cancer-specific genes in MSI colorectal adenocarcinoma (CRC) and MSI endometrial carcinoma (ECs) using a modified partial least squares discriminant analysis. We obtained a list of cancer-specific genes in MSI CRC and EC by taking the intersection of the genes obtained from tumor samples and normal samples. Specifically, we obtained publically available 1319 RNA sequencing data consisting of MSI CRCs, MSI ECs, normal colon including the rectum, and normal endometrium from The Cancer Genome Atlas and genome-tissue expression sites. To reduce gene-centric dimensions, we retained only 3924 genes from the original data by performing the usual differentially expressed gene screening for tumor samples using DESeq2. The usual partial least squares discriminant analysis was performed for tumor samples, producing 625 genes, whereas for normal samples, projection vectors with zero covariance were sampled, their weights were square-summed, and genes with sufficiently high values were selected. Gene ontology (GO) term enrichment, protein–protein interaction, and survival analyses were performed for functional and clinical validation. We identified 30 cancer-specific normal-invariant genes, including Zic family members (*ZIC1*, *ZIC4*, and *ZIC5*), *DPPA2*, *PRSS56*, *ELF5*, and *FGF18*, most of which were cancer-associated genes. Although no statistically significant GO terms were identified in the GO term enrichment analysis, cell differentiation was observed as potentially significant. In the protein–protein interaction analysis, 17 of the 30 genes had at least one connection, and when first-degree neighbors were added to the network, many cancer-related pathways, including MAPK, Ras, and PI3K-Akt, were enriched. In the survival analysis, 16 genes showed statistically significant differences between the lower and higher expression groups (3 in CRCs and 15 ECs). We developed a novel approach for selecting cancer-specific normal-invariant genes from relevant gene expression data. Although we believe that tissue-specific reactivation of embryonic genes might explain the cancer-specific differences of MSI CRC and EC, further studies are needed for validation.

## 1. Introduction

Microsatellite instability (MSI) is one of the main drivers in carcinogenesis in a wide range of tumors and a therapeutic target acting in a nearly cancer type-nonspecific way.^[1–7]^ Although the proportion of MSI-high (MSI-H) tumors is minimal in most tumor types, typically colorectal adenocarcinoma (CRC) and endometrial carcinoma (EC), these proportions are relatively high. The exact diagnosis of MSI is important because immune checkpoint inhibitors are more effective in these patients, irrespective of the histology or tissue of origin.^[2,8–11]^ Although mutational profiles differ between MSI and microsatellite-stable (MSS) tumors^[12]^ and MSI CRC has been reported to have a higher tumor mutation burden and lower copy number than MSS tumors,^[13]^ the differential organ-specific carcinogenesis initiated by MSI is not fully understood.

Obtaining a list of differentially expressed genes (DEGs) between different biological conditions using DNA microarrays or RNA sequencing (RNA-seq) is a routine analytic procedure in many research areas. In DEG analysis between MSI-H and MSS CRCs, the most frequently altered functional classes are cell cycle, DNA replication, recombination, repair, gastrointestinal disease, and immune response.^[14]^ MSI-H CRCs have been found to be characterized by a higher expression of immune-related genes compared with MSS CRCs.^[15]^ However, studies on DEG analysis between MSI-H and MSS CRCS are rare.

Although different MSI tumors, typically CRCs and ECs, are characterized by similar carcinogenesis, marked differences in prognosis and response to therapy have been identified.^[3,6,7,9–11]^ Unlike routine DEG analysis,^[16,17]^ cross-tissue DEG analysis such as the one between MSI CRCs and ECs is challenging.^[18]^ If routine DEG analysis procedures are applied between MSI CRCs and ECs, most of DEGs are normally functioning tissue-specific genes that are unrelated to carcinogenesis. To overcome these issues, in the Cancer Genome Atlas (TCGA) PanCancer project, methodologies based on orthogonal partial least squares discriminant analysis (OPLS-DA) have been attempted as alternatives to routine procedures.^[18]^ Many normal tissue-specific genes were removed. These results were hard to obtain if only standard DEG identification procedures, such as DeSeq2 or edgeR, were used.^[19,20]^

In our previous study, instead of directly selecting DEGs, we attempted functional profiling of the differences between MSI CRCs and ECs using biclustering and GO tree analysis.^[21]^ Although our approaches are more effective under heterogeneous conditions, direct selection of cancer-specific marker genes differently expressed between MSI CRCs and ECs is still necessary and synergistic with our approaches.

Partial least squares discriminant analysis (PLS-DA) is a statistical method in which spatially represented dependent variables (categorical) and independent variables are projected onto a new space to maximize the covariance between the projections.^[22]^ If a similar projection-based method is applied to continuous dependent variables, instead of categorical variables, it is called partial least square (PLS) regression. We found out that the PLS-DA procedures can be easily modified to select cancer-specific normal-invariant genes between MSI CRCs and ECs by modifying the directions of projections in accordance with our intent.

In this study, we developed a PLS-modified method to select cancer-specific and normal-invariant genes in MSI CRCs and ECs using gene expression data from TCGA and genotype-tissue expression (GTEx) data. Indeed, these genes were differentially expressed between MSI CRCs and ECs but were invariant to normal tissue types (colon and endometrium), which could not be achieved by routine DEG analysis. Functional analysis suggested distinct functions of these genes, and survival analysis validated the clinical significance.

## 2. Methods

### 2.1. Data collection and preprocessing

**Figure 1.**
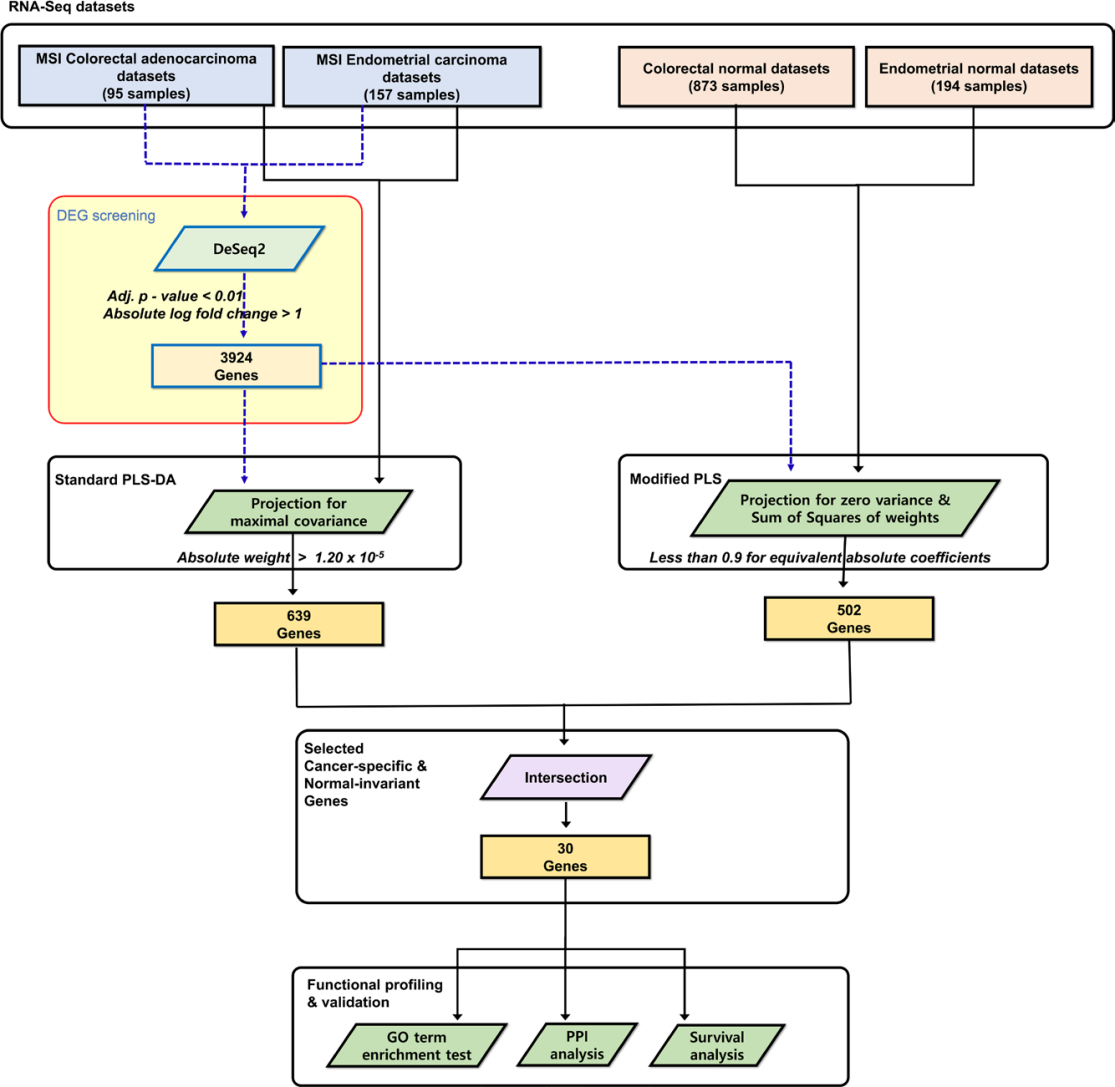
Overall workflow of the study. Methods or tools are represented as parallelograms and data or processed results as rectangles. Rounded rectangles are used for a more detailed description of a set of processes or data. Dashed lines are used for visual clearance. adj. *P* value = adjusted *P* value using false discovery rate, DEG = differentially expressed gene, DeSeq2 = R package, DESeq2, GO = gene ontology, PLS-DA = partial least square discriminant analysis.

The workflow of this study is illustrated in Figure 1. In this retrospective observational study, we have completed the strengthening the reporting of observational studies in epidemiology statement. Cleansed and refined RNA-seq data for TCGA CRC, EC, and GTEx of colon and endometrial samples were obtained and downloaded from the Recount3 project site, along with the corresponding clinicopathological data.^[23]^ As in our previous research,^[21]^ in accordance with the policies of TCGA and GTEx, neither ethical approval nor patient consent was required for this study. Although there are many sources of TCGA and GTEx data, the Recount3 data were more suitable to our research because the Recount3 project aims to provide uniformly processed analysis-ready RNA-seq data using optimized pipelines.^[23]^ Several studies have revealed that TCGA normal and GTEx samples can be seamlessly combined with minimal bias.^[18,21,23]^ Selection of MSI-H cancer and normal samples (colon and endometrium) yielded a total of 1319 samples: 873 normal colorectal tissue, 95 colorectal MSI cancers, 194 normal endometrial tissue, and 157 endometrial MSI cancers. As transcript per million values were used for PLS-DA, the directly obtained gene-sample count matrices were transformed into transcript per million matrices with the same gene-sample structure using external gene size information and read count information.^[24]^

### 2.2. Selection of cancer-specific and normal-invariant genes

Cancer-specific normal-invariant genes were selected by integrating the products of 2 main procedures, (1) usual PLS-DA and (2) PLS-modified procedures. (1) For a tumor gene expression matrix, *X*_T_ (*n* samples × *p* genes) and a cancer type vector *Y*_T_ (*n* samples), whose values are either CRCs or ECs, usual PLS-DA was performed and the genes were selected if their weight values of the first PLS component were large enough. (2) For a gene expression matrix of normal samples, *X*_N_ (*m* samples × *p* genes) and a normal tissue type vector *Y*_N_ (*m* samples), whose values are either colon (including rectum) or endometrium, PLS-modified procedures were performed to produce a squared sum of weight vectors of the genes and genes with sufficiently large values were selected. Finally, the genes were selected by taking the intersections of the 2 products (genes) from procedures (1) and (2). This final list of genes was expected to be cancer-specific and normal-invariant because most of normal tissue-specific genes would be filtered during the PLS-modified procedures.

When the *X*_T_ or *X*_N_ was composed of more than 20,000 protein-coding genes, the procedures, particularly procedure (2), were computationally infeasible and noisy. Therefore, we limited the number of genes in each matrix to less than 5000. As a gene-screening procedure, we performed DEG analysis between CRCs and ECs using DESeq2 with the threshold of absolute log fold change >1 and false discovery rate-adjusted *P* value <0.01.^[19]^ A total of 3924 genes were selected that were tolerable for the subsequent procedures. With the introduction of these DEG screening procedures, many of the noisy genes were expected to be filtered, biologically significant genes were retained, and all computation durations could be saved.

For the usual PLS-DA using *X*_T_ (252 samples × 3924 genes) as an input, the plsda function in mdatools was applied with a mean centering setting.^[25]^ Genes with weight values of the first component >1.2 × 10^−5^ were selected.

For *X*_N_ (1067 samples × 3924 genes), we developed a novel PLS-modified procedure to select genes whose expression values were relatively invariant to the tissue type (colon or endometrium). The main idea is that instead of the usual PLS projection, where the covariance is maximized between projected *X* and *Y*, *X* and *Y* are projected in a direction where the absolute covariance is minimized. Under these conditions, we can easily expect that the normal tissue type is indiscriminate between the colon and the endometrium. Specifically (1) the *X* and *Y* are mean-centered as 0, (2) *v* = *X*^T^*Y* is calculated, (3) a set of unit vectors, *w* is uniformly sampled from a set consisting of the infinite numbers of elements with possible values satisfying *vw* = 0 (each vector in *w* is perpendicular to *v*) and ∥*w*∥ = 1, (4) squared sum of *w* is computed to produce a vector of length *p*, (5) the genes with large enough values are selected.

In the procedure (3), vectors in *w* should be perpendicular to *v* because the covariance is zero under that condition, which indicates that projections are made so as not to differentiate between the normal colon and endometrium by gene expression. All unit vectors in *w* cannot be completely enumerated because they are infinite in numbers. Therefore, instead of enumerating, we uniformly sampled as many candidates as possible from an infinite number of possible candidates. These problems are geometrically equivalent to obtaining a large number of sample points from the intersection of the 3924 dimensional hypersphere of radius 1 and 3924 dimensional hyperplane both perpendicular to *v* and passing through the origin (0, 0, …, 0). The intersection was formulated as a 3923 dimensional hypersphere. For sampling points from the hypersphere (3923 dimensional), we used an orthonormal basis of 3924 dimensions where vector *v* is included. To obtain one among infinitely possible orthonormal bases, the Gram-Schmidt process was applied.^[26]^ With the resultant orthonormal basis (*b*_1_, …, *b*_3923_) (*v* excluded), all points in the intersection (a 3923 dimensional hypersphere) can be represented by the following formula.


$$\begin{array}{l} \begin{array}{llll} & b_{1}cos\phi_{1}+b_{2}sinf_{1}cos\phi_{2}+b_{3}sin\phi_{1}sin\phi_{2}cos\phi_{3}\; & +\ldots +b_{3922}sin\phi_{1}sin\phi_{2}\ldots \;cos\phi_{3922}\; & +b_{3923}sin\phi_{1}sin\phi_{2}\ldots \;sin\phi_{3922},\; \end{array} \end{array}$$

where 0 ≤ *ф*_1_ ≤ π, 0 ≤ *ф*_2_ ≤ π, 0 ≤ *ф*_3_ ≤ π, …, 0 ≤ *ф*_3921_ ≤ π, 0 ≤ *ф*_3922_ ≤ 2π.

In procedure (5), we identified a very strong negative correlation between the squared sums of the projection vectors and the absolute weights of *v*. Because our original procedures are too time-consuming (3,500,000 samplings for 24 hours even under high-performance graphics processing unit) and numbers close to zeros were multiplied or added too much time, for stability and reproducibility, the coefficients of *v* seemed to be more appropriate in gene selection. Therefore, in the actual implementations, the genes with absolute coefficients of *v* < 0.9 were selected instead of using squared sums as selection metrics. We will consider presenting analytic proof for such properties of *v* and *w* in future publications.

### 2.3. Visualization of selected genes and gene ontology (GO) term enrichment test

We visualized the gene expression of cancer-specific normal-invariant genes to visually inspect whether the selected genes were indeed cancer-specific and normal-invariant. The bioconductor package pheatmap was used.^[27]^

To identify enriched gene ontology (GO) terms for these genes, a GO term enrichment test was conducted using the Database for Annotation, Visualization, and Integrated Discovery.^[28]^

### 2.4. Protein–protein interaction (PPI) analysis

To identify physical or functional interactions among the cancer-specific normal-invariant genes, we performed PPI analysis using the Search Tool for the Retrieval of interacting Genes/Proteins (STRING) database.^[29]^ Using rba_string_network_image and rba_string_enrichment_ppi functions in that package, the network of the genes was visualized, and PPI-based functional enrichment test was performed. The same functions were performed after expanding the previous network by adding one-degree neighbors.

### 2.5. Survival analysis

To evaluate the functional significance of cancer-specific normal-invariant genes, we performed a survival analysis using TCGA clinical data for CRCs and ECs. For each gene, according to the distribution of the sample-wise gene expression, the samples were dichotomized into high- and low-expression groups, and the sample information was used as patient information. Survival analyses were independently conducted for CRCs and ECs. In the survival analysis, the overall survival was compared between high- and low-expression cases.

## 3. Results

### 3.1. Evaluation of the procedures

**Figure 2.**
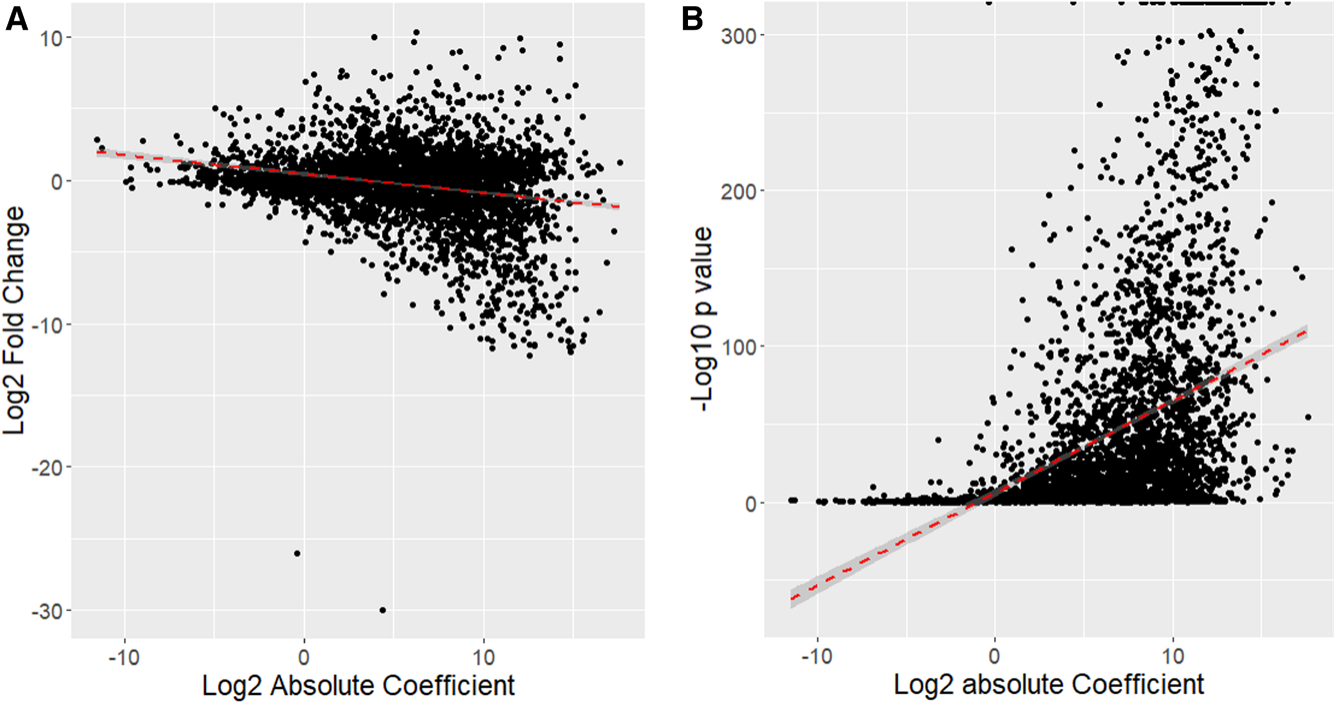
Comparison between the results obtained from modified PLS and standard DEG analysis (DESeq2). Because coefficients of an orthogonal vector are the main results of the modified PLS procedure, and log2 fold change and *P* values are the main results of the DESeq2 for normal samples, the comparisons are represented as 2 scatter plots, one for coefficients of the orthogonal vector and log2 fold change (A) and the other for coefficients of the orthogonal vector and *P* values (B). Each dot in the plots represents a gene (3924 points in each plot). Dashed red lines indicate linear regression results. DEG = differentially expressed gene, PLS = partial least square.

We evaluated the modified PLS procedure applied to normal samples (explained in section 2.2) by comparing the results obtained by DESeq2 (Fig. 2). DeSeq2 results were obtained by comparing 873 normal colorectal tissue and 194 normal endometrial tissue. When the coefficients in the modified PLS and log2 fold change of DESeq2 were compared, there was a strong inverse relationship between them, despite the large variability (Fig. 2A). We also compared the relationships of the *P* values obtained from DESeq2 and found inverse relationships between them (Fig. 2B). Because normal-invariant genes are expected to show lower fold changes and high *P* values, these results demonstrate that our modified PLS procedure works as intended.

We then tested whether cancer-specific normal-invariant genes could be obtained using standard DEG analysis tools such as DESeq2, without using the procedures we developed. When we applied log2 fold change <0.5 and adjusted *P* value >0.6, we identified 640 genes. However, these 640 genes were completely different from the 30 genes identified using our procedures. Only 5 genes overlapped, indicating that it was difficult to obtain these 30 genes using any other approach.

### 3.2. Selection of cancer-specific and normal-invariant genes

**Figure 3.**
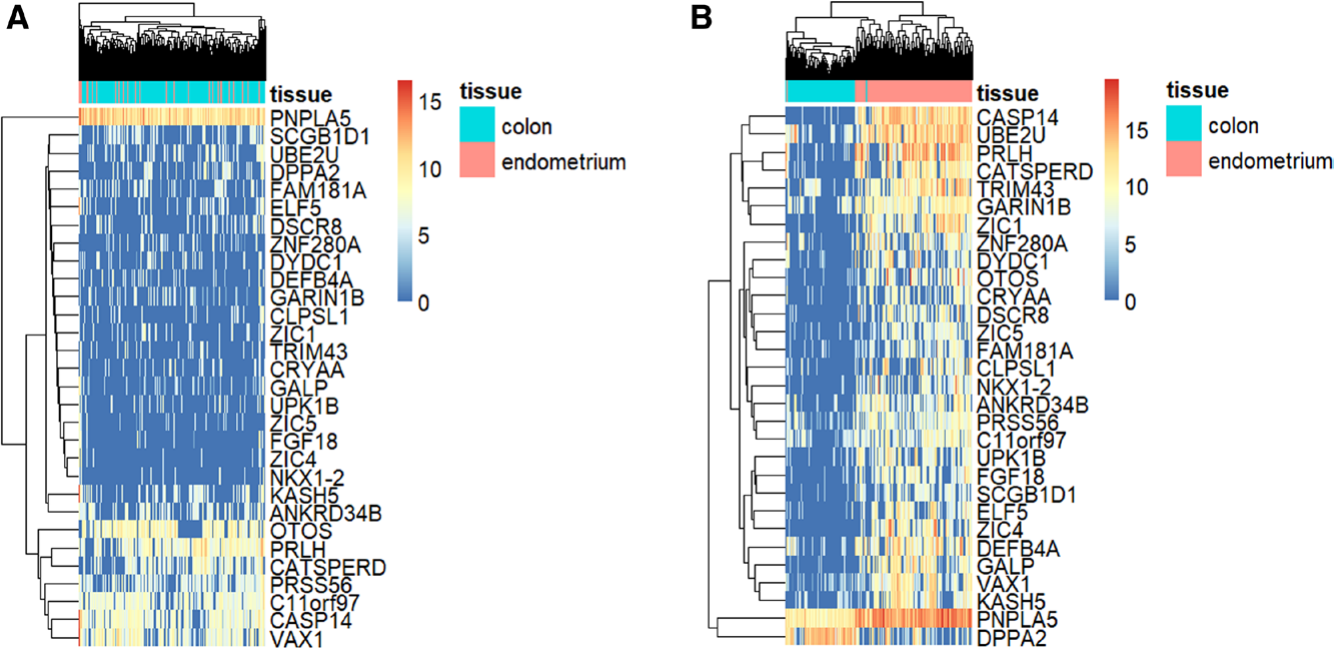
Comparison of gene expression (nonscaled) of 30 cancer-specific normal-invariant genes between normal (A) and tumor samples (B). The tissue type (colon and endometrium) at the right side of each heatmap indicates normal colon and endometrium in the normal tissue (A) and MSI CRC and MSI EC in tumor (B), respectively.

**Figure 4.**
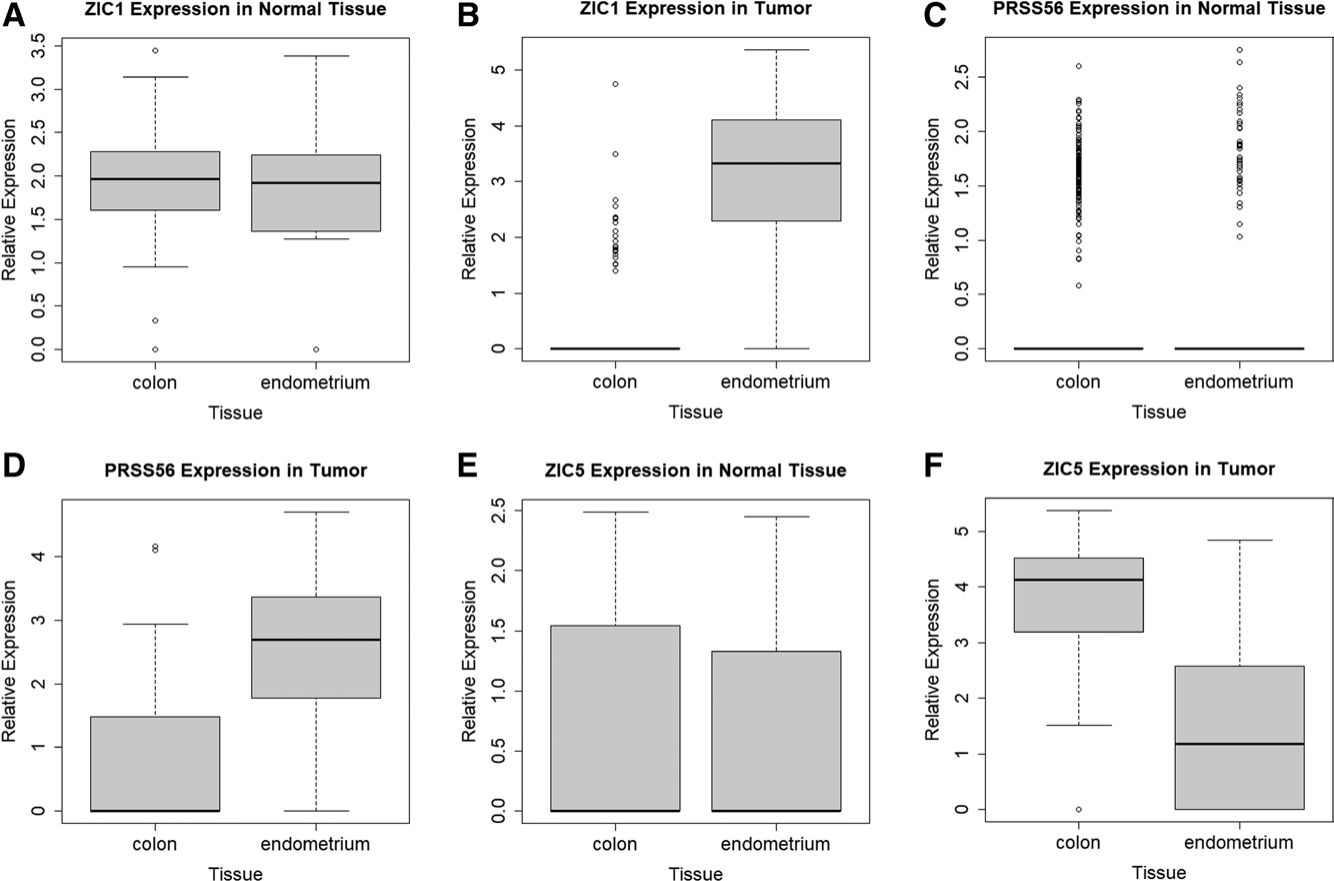
Relative gene expression levels of individual genes, as exemplified by *ZIC1* (A and B), *PRSS56* (C and D), and *ZIC5* (E and F) in the normal (A, C, and E) and tumor (B, D, and F) samples. The colon and endometrium at the *x* axis in each boxplot indicate normal colon and endometrium in the normal tissue (A, C, and E) and MSI CRC and MSI EC in tumor (B, D, and F), respectively. CRC = colorectal adenocarcinoma, EC = endometrial carcinoma, MSI = microsatellite instability.

Using PLS-DA and modified PLS procedures (explained in Section 2.2.), we identified 30 cancer-specific normal-invariant genes, which were the intersections of 625 and 502 genes obtained from the 2 independent procedures, respectively (Table 1 and Fig. 3). When tumor and normal samples were clustered with these genes, tumor samples were almost purely clustered according to the tissue of origin, whereas normal samples were almost homogeneously mixed with each other, indicating that these genes indeed have cancer-specific normal-invariant properties (Fig. 3A and B). When expression levels were investigated at the individual gene level, tumor samples showed more pronounced differences between tissue origins than normal samples (Fig. 4A, C, and E for normal samples, and Fig. 4B, D, and F for tumor samples).

**Table 1:** Selected cancer-specific normal-invariant genes and their functions.

Gene symbol	Description	Major functions mainly associated with cancer	Orthogonal vector weight (coefficient) obtained from normal samples	Weight obtained from tumor sample (×10^−5^)
*PRLH*	prolactin-releasing hormone	Enriched in EC^[30]^	−0.02	1.27
*PNPLA5*	Patatin like phospholipase domain containing 5	Enriched in thyroid cancer^[31]^	0.03	1.37
*CASP14*	Caspase 14	Enriched in breast cancer^[32]^	0.03	1.28
*GARIN1B*	Golgi associated RAB2 interactor 1B	Involved in sperm head formation^[33]^	−0.04	1.38
*ELF5*	E74-like ETS transcription factor 5	Inhibits the proliferation and invasion of breast cancer^[34]^	0.04	1.3
*ZIC5*	Zic family member 5	Contributes to the aggressiveness of prostate cancer^[35]^	0.06	1.32
*FAM181A*	Family with sequence similarity 181 member A	Enriched in glioma^[36]^	0.09	1.85
*TRIM43*	Tripartite motif containing 43	May induce ubiquitin-mediated degradation of oncogenic products^[37]^	−0.11	1.48
*VAX1*	Ventral anterior homeobox 1	Prognostic biomarker in bladder cancer^[38]^	−0.12	1.43
*ZIC1*	Zic family member 1	Tumor suppressor in breast cancer^[39]^	−0.12	2.07
*FGF18*	Fibroblast growth factor 18	Prognostic marker in various cancers^[40]^	−0.17	1.78
*CRYAA*	Crystallin alpha A	Enriched in retinoblastoma^[41]^	0.18	2.01
*KASH5*	KASH domain containing 5	Associated with meiotic arrest^[42]^	−0.24	1.83
*DPPA2*	Developmental pluripotency associated 2	Highly expressed in stem cells and activated in cancer cells^[43]^	−0.25	1.22
*SCGB1D1*	Secretoglobin family 1D member 1	Possibly act as tumor suppressor^[44]^	0.26	1.21
*ZNF280A*	Zinc finger protein 280A	Promotes proliferation in CRC^[45]^	0.33	1.26
*DYDC1*	DPY30 domain containing 1	Associated with poor prognosis in 10q loss neuroblastoma^[46]^	0.37	-1.83
*DEFB4A*	Defensin beta 4A	Prognostic biomarker in CRC^[47]^	0.37	1.41
*CATSPERD*	Cation channel sperm associated auxiliary subunit delta	Suppressed in oral cancers^[48]^	−0.41	1.25
*ZIC4*	Zic family member 4	Contributes to cancer progression in hepatocellular carcinoma^[49]^	−0.41	1.46
*UBE2U*	Ubiquitin-conjugating enzyme E2 U	Provoke various cancers^[50]^	−0.44	2.11
*OTOS*	Otospiralin	Associated with cisplatin-induced ototoxicity^[51]^	0.49	1.54
*ANKRD34B*	Ankyrin repeat domain 34B	Enriched in prostate cancer and renal cell carcinoma^[52]^	0.57	1.68
*GALP*	Galanin like peptide	Enriched in neuroblastoma^[53]^	−0.6	1.2
*DSCR8*	Down syndrome critical region 8	Promote the proliferation of hepatocellular carcinoma^[54]^	−0.6	1.21
*CLPSL1*	Colipase like 1	Associated with lymphatic metastasis in CRC^[55]^	−0.6	1.44
*NKX1-2*	NK1 homeobox 2	Transcriptional repressor promoting carcinogenesis^[56]^	−0.6	1.52
*PRSS56*	Serine protease 56	Enriched in gastric carcinoma and CRC^[57]^	−0.75	3
*UPK1B*	Novel chromosome 3 open reading frame 30 (C3orf30) and uroplakin 1B (UPK1B)	Regulator of Wnt/β-catenin pathway^[58]^	−0.77	1.25
*C11orf97*	Chromosome 11 open reading frame 97	Uncharacterized protein	−0.79	2.49

CRC = colorectal adenocarcinoma, EC = endometrial carcinoma.

### 3.3. Functional profiling using GO term enrichment test

We investigated the functional features of the cancer-specific normal-invariant genes using GO terms. Although not statistically significant, GO biologic process terms, such as central nervous system development, cell differentiation, regulation of transcription by RNA polymerase, camera-type eye development, and response to insulin, were enriched (Table 2). When only normal-derived 502 genes or only tumor-derived 625 genes were independently profiled, none of the GO biologic process terms were identified, indicating that very distinct functions were retained among the 30 genes.

**Table 2:** Enriched GO biologic process (BP) terms of 30 refined genes.

Term	Description	Fold enrichment	*P* value	False discovery rate
GO:0007417	Central nervous system development	23.33	<.001	0.07
GO:0030154	Cell differentiation	6.06	.007	0.36
GO:0006357	Regulation of transcription by RNA polymerase II	3.58	.009	0.36
GO:0043010	Camera-type eye development	39.15	.048	1.00
GO:0032868	Response to insulin	21.64	.085	1.00

GO = gene ontology.

When we more deeply inspected on the cell differentiation, though *VAX1* and *PRLH* were not directly annotated to GO:0030154 (cell differentiation), these genes were found to be apparently associated with cell differentiation. These findings suggest that if slightly different annotation schemes were applied to the official GO annotation, cell differentiation may be enhanced.

### 3.4. Protein–protein interaction (PPI) analysis

**Figure 5.**
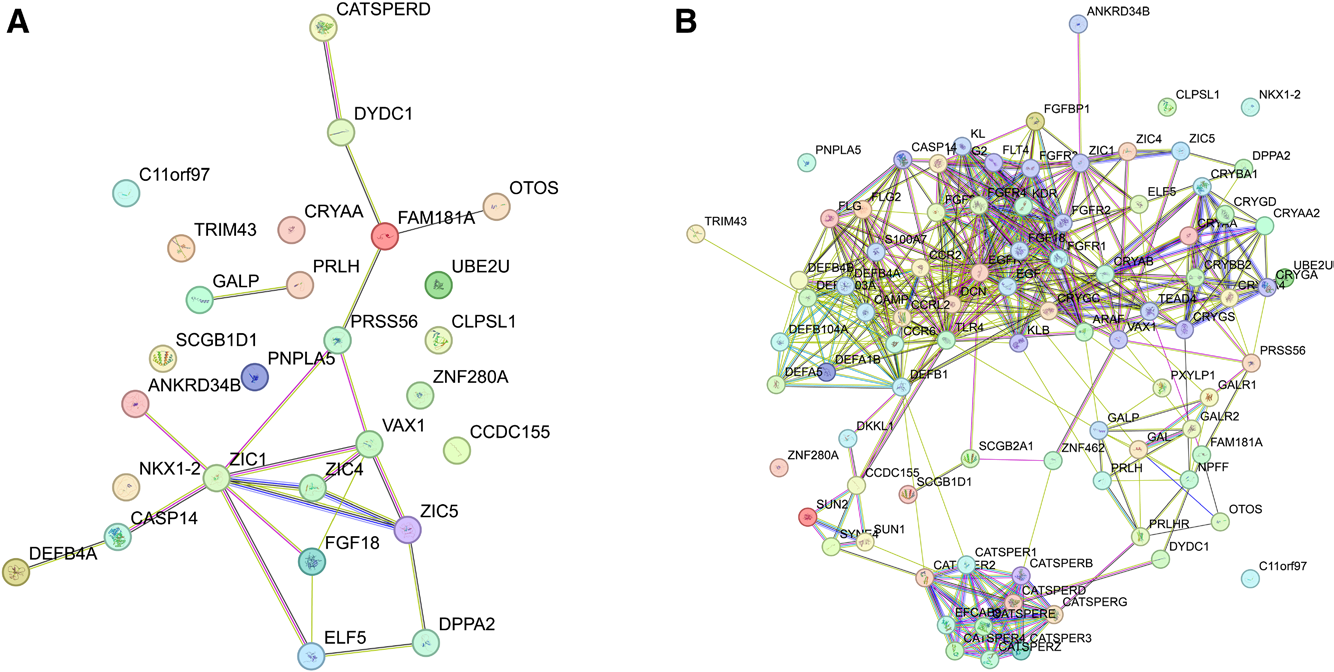
Network representation of protein–protein interaction (PPI) of 30 cancer-specific normal-invariant genes (A) and an expanded network with the degree neighbors of the 30 genes added to the existing one (B). The nodes represent genes and the edges represent the interactions between them. Different edge colors represent various types of interactions.

To investigate whether there were any known physical and functional relationships among cancer-specific normal-invariant genes, we analyzed the STRING database network (Fig. 5A). Among the 30 genes, 27 genes mapped to the STRING network (excluding *GARIN1B*, *DSCR8*, and *UPK1B*) (Fig. 5A). In the network, 17 genes have at least one connected genes, whereas 10 genes, including *OTOS*, *C11orf97*, and *UBE2U*, were solitary. The connected genes were clustered into 2 groups: one with *GALP* and *PRLH* and the other with 15 genes, including *ZIC1*, *ZIC5*, and *ELF5*. The network was enriched in IPR041643 (ZIC protein, zinc finger domain in InterPro database, false discovery rate = 0.00092).^[59]^

We attempted to expand the previous network by adding the first-degree neighbors (Fig. 5B). A total of 62 nodes were added to the previous network of the 27 genes. Two of the previous solitary nodes (*OTOS* and *CCDC155*) became connected nodes by adding neighbors, and the connections between the nodes of the previous network became denser. In the functional enrichment test, the expanded network was enriched in many cancer-associated pathways such as Rap1, MAPK, Ras, PI3K-Akt, and NOD-like receptor signaling pathways.

### 3.5. Survival analysis

We assessed the clinical utility of 30 cancer-specific normal-invariant genes by survival analysis. Survival analysis was conducted independently for each CRC or EC cohort. For each pair of 30 genes and cohorts (CRCs and ECs), the patients were divided into 2 groups, a higher expression group and a lower expression group, depending on the expression level within the cohort, resulting in 60 (30 × 2) survival analyses.

**Figure 6.**
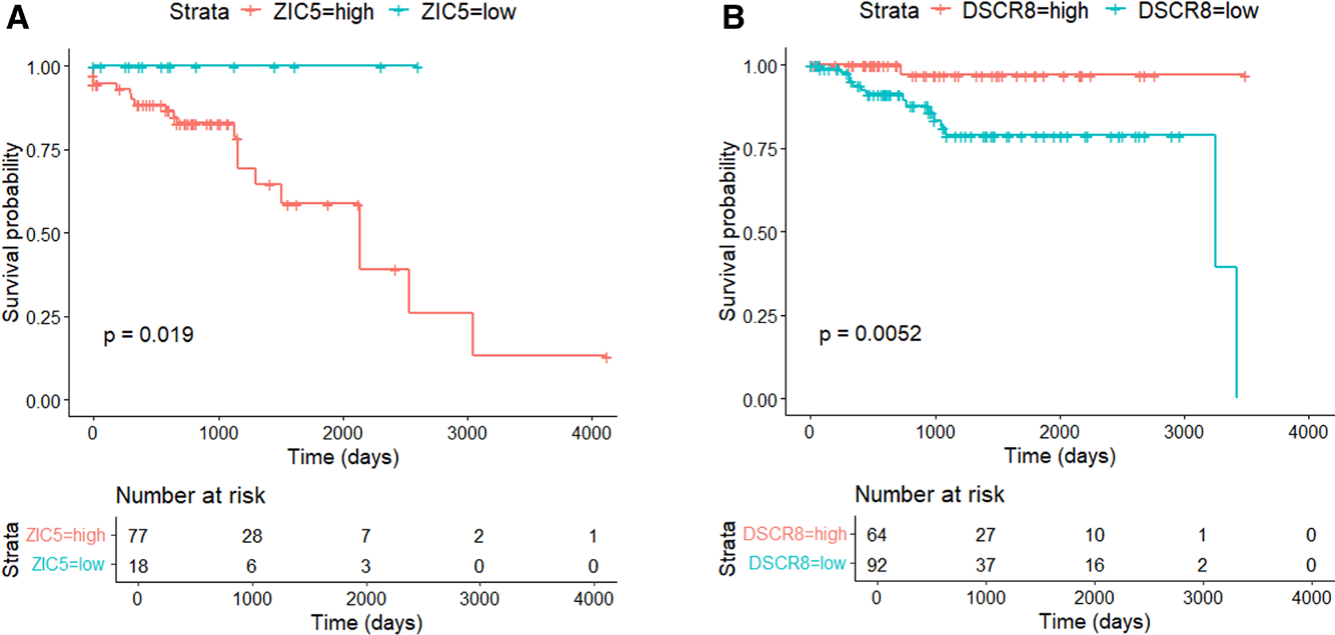
Survival analysis of 2 representative genes (*ZIC5* and *DSCR8*) in the CRC (A) and EC (B) cohort, respectively. The patients in each cohort are dichotomized into high-expression and low-expression groups according to their expression levels. CRC = colorectal adenocarcinoma, EC = endometrial carcinoma.

Among the 30 genes, 16 were significantly different between the lower and higher expression groups (3 in CRCs and 15 ECs) (Fig. 6A and B).

## 4. Discussion

In this study, we attempted to identify cancer-specific genes differentially expressed between MSI CRCs and ECs, we developed a methodology to discover more cancer-specific genes by retaining genes with cancer type-specific gene expression patterns and efficiently excluding cancer-unrelated confounder genes. These schemes are based on the fundamental insights that genes with cancer-specific properties cannot be selected by considering only tumor samples. By applying the core idea of PLS-DA to the discovery of cancer-specific genes, concrete methodological schemes can be developed. We also identified 30 cancer-specific normal-invariant genes, which would contribute to expanding our knowledge of the differences between MSI CRCs and ECs in carcinogenesis.

To refine cancer-specific normal-invariant genes, gene expression data from at least 4 types of samples should be prepared. A typical example is the comparison of cancers originating from 2 different organs when reference normal tissue data are available. Generally, standard DEG identification methods are widely applicable to many biologically significant issues; however, when comparing cancers from 2 different organs, too many normal tissue-specific genes are mixed with cancer-specific genes. By applying the aforementioned scheme to these situations, more refined cancer-specific genes can be identified by retaining only normal-invariant genes. Our schemes are also applicable beyond the oncology field only if samples of the main interest and reference samples with no interest are provided together.

As in Table 1, most of the 30 cancer-specific normal-invariant genes were associated with tumorigenesis; however, some of them are worth mentioning. The human zinc finger of the cerebellum (ZIC) family of genes are essential genes during development.^[60]^ In various cancers, including melanoma, glioma, gastric cancer, CRCs, breast cancer, and pancreatic cancers, the significances of ZICs have been increasingly identified.^[61–68]^ ZIC proteins act as transcriptional regulators during organ development.^[69]^ In CRCs, *ZIC5* can stimulate cancer cell proliferation via the CDK1/CDC25c pathway, whereas depletion of *ZIC5* reveals the opposite antineoplastic effects.^[70]^ We identified 3 ZICs (*ZIC1*, *ZIC4*, and *ZIC5*) as differentially expressed cancer-specific genes. In accordance with the previous studies, patients with higher expression of *ZIC5* showed a worse prognosis than those with lower expression groups in MSI CRCs, although the presence of MSI-specific effects of *ZIC5* should be further investigated (Fig. 6A).

Developmental pluripotency factors 2 and 4 (*DPPA2* and *DPPA4*) are transcriptional regulators that interact with chromatin-modifying complexes in pluripotent stem cells.^[43,71,72]^ Reactivation of *DPPA2* and *DPPA4* is associated with many types of cancers.^[71]^ In CRCs, *DPPA2* mRNA expression has been reported to be significantly correlated to the advanced stage.^[73,74]^ However in our survival analysis using TCGA gene expression data, statistically significant survival differences were not identified between the higher expression and lower expression groups.

*PRSS56*, a serine protease with trypsin-like activity, is a novel cancer-testis antigen that plays an important role in eye development and neurogenesis.^[75]^ In humans, mutations in this gene cause an autosomal recessive developmental disorder known as posterior microphthalmos.^[76–78]^ *PRSS56* can be reactivated in various cancers and enriched in CRCs, ECs, cervical cancer, melanoma, and urothelial carcinoma.^[57,79]^ In CRCs and gastric carcinoma, *PRSS56* is activated by the hypomethylation of promoter regions and activated gene products promote cancer progression via the PI3K/Akt axis.^[57]^ In our analysis, *PRSS56* was more highly expressed in MSI ECs than in MSI CRCs. In survival analysis, higher expression of *PRSS56* was associated with better overall survival than lower expression (*P* = .012). These findings in MSI ECs are not consistent with previous reports on CRCs because, in CRCs, *PRSS56* appeared to promote tumor progression.^[57]^ However, the high immunogenicity of the cancer-testis antigen may explain part of this discrepancy. Considering that there are few researches on the roles of *PRSS56* in ECs (not MSI ECs alone), more research is required. The other significant features of the 30 genes regarding carcinogenesis are listed in Table 1.^[30–42,44–56,58]^

Based on the above findings, the following hypothesis was proposed: some of the 30 cancer-specific normal-invariant genes may be reactivated embryonic genes that became inactive after terminal differentiation and this reactivation might be tissue-specific. The role of MSI in this process seems nonspecific and the distinguishing features of these MSI cancers might be similar to those of their MSS counterpart in an organ-specific way. We believe that our hypothesis is worth investigating and further studies are necessary to confirm it.

Although we presented a list of 30 cancer-specific genes that seem reasonable in the context of colorectal and endometrial carcinogenesis, our approach has some limitations. First, we obtained only 30 genes, which seemed too small to identify enriched pathways or GO terms. The adoption of more flexible approaches focusing on the relative contribution of genes between normal and tumor samples may yield more reasonable numbers of genes with decreased false positives. Second, we selected genes based mainly on the weight of the projection vector, which could be very large or small by chance. Confidence interval-like metrics are helpful for selecting genes based on more stable criteria. One simple approach is to apply bootstrapping-based approaches to produce confidence intervals for the weights. Third, we could not evaluate the robustness of our approaches on the sample size and dominance of one class because simulation studies for this are too time-consuming. These issues will be addressed in more detail in future studies. However, the influence of the sample size and class bias would not be large in our research because the TCGA data have a large number of samples and are not biased into one class too much.

In conclusion, to refine unique features of MSI CRCs or ECs that are not confounded by normal tissue-specific genes, we developed novel methodology schemes based on the core concept of PLS and identified 30 cancer-specific normal-invariant genes with unique features in carcinogenesis. Our approach can be applicable to any field beyond oncology only if data simultaneously representing conditions of interest and reference are available. The 30 cancer-specific normal-invariant genes would provide insights into how the same MSI cancers have different therapeutic responses and prognoses depending on whether they originate from the colon or endometrium. The role of these genes would be clearer if the same research is extended to other types of MSI cancers, such as those in the stomach, small intestine, and hepatobiliary tract.

## Author contributions

**Conceptualization:** Young Soo Song, Woong Na, Sung Hak Lee.

**Formal analysis:** Young Soo Song, Woong Na, Sung Hak Lee, Seunghee Lee.

**Funding acquisition:** Young Soo Song.

**Methodology:** Young Soo Song, Woong Na, Sung Hak Lee, Seunghee Lee.

**Project administration:** Young Soo Song, Yong Min Kim, Mihye Kwon.

**Visualization:** Young Soo Song, Woong Na, Sung Hak Lee, Jong-Seok Kim, Seung Yun Han, Yong Min Kim, Mihye Kwon.

**Writing—original draft:** Young Soo Song, Woong Na, Sung Hak Lee.

**Writing—review & editing:** Young Soo Song, Woong Na, Sung Hak Lee, Seunghee Lee, Jong-Seok Kim, Seung Yun Han, Yong Min Kim, Mihye Kwon.

**Investigation:** Woong Na, Sung Hak Lee, Jong-Seok Kim.

**Validation:** Sung Hak Lee, Mihye Kwon.

**Software:** Seunghee Lee, Mihye Kwon.

**Resources:** Seung Yun Han, Mihye Kwon.

**Supervision:** Mihye Kwon.
